# The Search for an Effective Therapy to Treat Fragile X Syndrome: Dream or Reality?

**DOI:** 10.3389/fnsyn.2017.00015

**Published:** 2017-11-06

**Authors:** Sara Castagnola, Barbara Bardoni, Thomas Maurin

**Affiliations:** ^1^Université Côte d’Azur, CNRS, Institut de Pharmacologie Moléculaire et Cellulaire (IPMC), Valbonne, France; ^2^Université Côte d’Azur, INSERM, CNRS, Institut de Pharmacologie Moléculaire et Cellulaire (IPMC), Valbonne, France

**Keywords:** Fragile X Syndrome, *FMR1*, ASD, MMP-9, IGF-1, endocannabinoid system, oxytocin, serotonin

## Abstract

Fragile X Syndrome (FXS) is the most common form of intellectual disability and a primary cause of autism. It originates from the lack of the Fragile X Mental Retardation Protein (FMRP), which is an RNA-binding protein encoded by the Fragile X Mental Retardation Gene 1 (*FMR1*) gene. Multiple roles have been attributed to this protein, ranging from RNA transport (from the nucleus to the cytoplasm, but also along neurites) to translational control of mRNAs. Over the last 20 years many studies have found a large number of FMRP mRNA targets, but it is still not clear which are those playing a critical role in the etiology of FXS. So far, no therapy for FXS has been found, making the quest for novel targets of considerable importance. Several pharmacological approaches have been attempted, but, despite some promising preclinical results, no strategy gave successful outcomes, due either to the induction of major side effects or to the lack of improvement of the phenotypes. However, these studies suggested that, in order to measure the effectiveness of a specific treatment, trials should be redesigned and new endpoints defined in FXS patients. Nevertheless, the search for new therapeutic targets for FXS is very active. In this context, the advances in animal modeling, coupled with better understanding of neurobiology and physiopathology of FXS, are of crucial importance in developing new selected treatments. Here, we discuss the pathways that were recently linked to the physiopathology of FXS (mGluR, GABAR, insulin, Insulin-like Growth Factor 1 (IGF-1), MPP-9, serotonin, oxytocin and endocannabinoid signaling) and that suggest new approaches to find an effective therapy for this disorder. Our goal with this review article is to summarize some recent relevant findings on FXS treatment strategies in order to have a clearer view of the different pathways analyzed to date emphasizing those shared with other synaptic disorders.

## Introduction

### Fragile X Syndrome

Fragile X syndrome (FXS) is the most common form of inherited intellectual disability (ID) and a primary genetic cause of autism. Due to its X-linked nature, it occurs in around 1:4000 males and 1:7000 females and one in three FXS patients display Autism Spectrum Disorders (ASD). Furthermore, the syndrome is characterized by delay in development and intellectual capacity, which impairs cognitive, executive and language performance. The severity of the symptoms can be very different among patients, and some of the most common features of the disease include repetitive behaviors, hyperactivity, anxiety, mood disorders and epilepsy (Hagerman et al., 2009). At the cellular level, there are abnormalities in neuronal maturation and pruning (Bassell and Warren, 2008; Scotto-Lomassese et al., 2011). In the brain of FXS patients as well as in FXS animal models (*Fmr1*-KO mouse and d*FMR1-KO* flies), dendritic spines have an altered morphology, and they appear longer, thinner and more tortuous than normal (Bassell and Warren, 2008). The morphological abnormalities are associated with deregulated synaptic plasticity: in mouse hippocampal mGluR dependent Long Term Depression (LTD) is increased (Huber et al., 2002), while Long Term Potentiation (LTP) is reduced in somatosensory and anterior cingulate cortex (Desai et al., 2006) as well as in amygdala (Suvrathan and Chattarji, 2011) and in the CA1 region (Lauterborn et al., 2007; Seese et al., 2012).

FXS arises from a mutation in a single gene called Fragile X Mental Retardation Gene 1 (*FMR1*). The 5′ UTR of *FMR1* contains a CGG trinucleotide repeat that is polymorphic in the population. Once the repeats exceed 200 in number, methylation of the promoter is triggered, and this in turn causes the lack of expression of the gene and translation of its encoded protein, the Fragile X Mental Retardation Protein (FMRP; Bardoni et al., 2000). FMRP is an RNA-binding protein involved in different steps of mRNA metabolism, such as translational control (in soma and dendritic spines) and RNA transport (Maurin et al., 2014).

### Therapeutic Strategies for FXS

Exploration of the physiopathology of FXS as well as the search for targets of FMRP have been very active over the last 20 year. These targets include mRNAs and proteins. Most proteins interacting with FMRP are RNA-binding protein components of FMRP-containing ribonucleoproteic particles. However, interactors of FMRP are also ion channels, molecular motors and proteins involved in cytoskeleton remodeling pathways (Menon et al., 2004; Bardoni et al., 2006; Davidovic et al., 2007; Abekhoukh and Bardoni, 2014; Maurin et al., 2014, 2015; Ferron, 2016; Abekhoukh et al., 2017; Bienkowski et al., 2017). This effort resulted in the identification of multiple putative targets for the treatment of FXS. However, so far no efficient therapy is available for this disorder (reviewed in Maurin et al., 2014). For instance, the imbalance between excitatory and inhibitory systems in FXS has been known for 20 years, and it has been thought to be the key target for therapy. Indeed, the first ever therapeutic strategies to be tested for FXS in clinical trials targeted the glutamatergic system (the excitatory pathway known to be up-regulated in FXS; Bear et al., 2004; Berry-Kravis et al., 2016) and the γ-aminobutyric acid (GABA) system (the inhibitory pathway also dysregulated in the disease; D’Antuono et al., 2003).

The mGluR theory regarding the pathophysiology of FXS states that the lack of FMRP hyperactivates the mGluR5-mediated pathway leading to the most prominent features of FXS (Bear et al., 2004). Even if the molecular reasons of this exaggerated activation are not completely clear, many efforts have been done based on this theory in order to develop an effective strategy for both pharmacological and genetic rescue through the inhibition of the mGluR pathway (Bear et al., 2004). In the mammalian FXS animal model (*Fmr1*-KO mouse), the treatment with specific antagonists of the mGluR5 resulted in the rescue of cellular (dendritic spine morphology), synaptic (exaggerated LTD) and behavioral defects, but these successes were not translated to the human treatments. Indeed, no beneficial effects of mavoglurant (AFQ056, a previous mGluR5 well-characterized antagonist) were observed in a 12-weeks, double-blind study involving a large cohort of adolescent and adult FXS patients (Bailey et al., 2016; Berry-Kravis et al., 2016). A similar new clinical trial was performed in a cohort of 183 FXS using basimglurant, a potent and selective mGluR5-negative allosteric modulator (NAM), already administrated as a treatment of depression (Quiroz et al., 2016). Again, according to the evaluation of the ADAMS score, no improvement in patients’ behavior was observed (Youssef et al., 2017).

GABA is the main inhibitory neurotransmitter of the Central Nervous System (CNS). Based on an altered expression of GABA receptor subunits in the absence of FMRP (Adusei et al., 2010) and a reduced production of GABA (Davidovic et al., 2011), it has been shown that an impairment of the GABAergic system is involved in FXS (D’Hulst and Kooy, 2007; Braat et al., 2015). Direct administration of GABA to the patients is not possible, partly because of its poor brain penetration. Thus, to restore the normal inhibition rate of the CNS and consequently reverse some major phenotype features of the disease, investigators have tried to treat FXS patients with GABA receptor agonists. So far, compounds like Acamprosate, Ganaxolone, Arbaclofen and Riluzole have been tested, but, despite the very mild side effects and good tolerability, they have caused very limited improvements in preliminary studies (Berry-Kravis et al., 2012; Erickson et al., 2014a,b; Ligsay et al., 2017).

Besides Glutamate and GABA, other deregulated pathways have been identified in FXS. Recent pre-clinical studies revealed that modulating other signaling pathways could ameliorate FXS symptoms in the mouse model of FXS. Our goal with this review is to summarize some recent relevant findings on FXS treatment strategies, focusing on promising molecular pathways that are altered not only in FXS but also in other forms of synaptic disorders that might lead to the discovery of a future treatment for neurodevelopmental diseases. In addition to those described here, other therapeutic targets for future treatment of FXS can be considered, for example, Amyloid Precursor Protein (APP), Brain-Derived Neurotrophic Factor (BDNF), cAMP and *N*-methyl-D-aspartate (NMDA) receptor as illustrated in several recent reviews (Wei et al., 2012; Castrén and Castrén, 2014; Androschuk et al., 2015; Tian et al., 2015; Westmark et al., 2016).

### Insulin and Insulin-Like Growth Factor 1 Pathway

Transcriptomic analysis of cultured hippocampal neurons obtained from WT and *Fmr1*-KO embryos showed that at the top of the list of enriched gene expression pathways was the “Insulin signaling pathway” (Prilutsky et al., 2015). In *dFMR1-KO* flies insulin signaling is altered due to an elevated expression of *Drosophila insulin-like peptide 2* (*Dilp2*) gene in the insulin-producing cells (IPCs) of the brain. Administration of metformin, an FDA-approved anti-diabetic drug, to d*FMR1-KO* flies (Viollet et al., 2012), leads to an amelioration of memory defects (Monyak et al., 2017). In this context it is interesting to notice that recent studies have clearly demonstrated the improvement of different *in vitro*/*in vivo* hallmarks in *Fmr1*-KO mice (Gantois et al., 2017) and in seven FXS patients (Dy et al., 2017) using metformin treatment. The precise mechanism of action of metformin has not been deciphered yet, but it has been shown that this drug inhibits the mitochondrial respiratory-chain, specifically at the complex 1 level, without affecting any other steps of the mitochondrial machinery. This leads to a reduction in proton-driven synthesis of ATP from ADP and inorganic phosphate. In addition, through AMPK-dependent and -independent regulation, metformin can lead to the inhibition of glucose production by disrupting gluconeogenesis gene expression (Viollet et al., 2012). In *Fmr1*-KO mice, metformin treatment inhibited the mTORC1 and ERK pathways, that resulted in the compensation of up-regulated translation, a hallmark of FXS (Maurin et al., 2014; Gantois et al., 2017). Indeed, in *Fmr1*-null neurons an increased level of phosphorylation of the serine/treonine kinase S6K1 has been observed. S6K1 is a common target of the mTORC1 and ERK pathways that are deregulated in the absence of FMRP (Sharma et al., 2010; Bhattacharya et al., 2012; Gross and Bassell, 2014; Gross et al., 2015a,b). It is interesting to notice here that two inhibitors of S6K1 (PF-4708671 and FS-115) were used in preclinical studies in mouse improving aberrant social interaction and behavioral inflexibility in Y-maze (for review see Gross and Bhattacharya, 2017). On the other side, also reducing the activation of the ERK pathway with lovastatin (a drug that inhibits the Ras-ERK1/2 activation by interfering with Ras recruitment to the membrane) or rimonabant (see endocannabinoid pathway) resulted in an improvement of *Fmr1-KO* cognition (Busquets-Garcia et al., 2013; Osterweil et al., 2013).

Obesity is often a co-morbid issue observed in FXS patients (Tounian et al., 1999; Raspa et al., 2010). Importantly, metformin has been used to treat seven obese FXS patients who showed improved cognition, language behavior along with obesity condition (Dy et al., 2017) proving to be an effective treatment for the FXS patients.

Insulin-like Growth Factor 1 (IGF-1) is a hormone primarily secreted by hepatocytes in response to Growth Hormone (GH) and, like insulin, promotes a decrease of glycaemia. IGF-1 promotes anabolic processes and tissue growth throughout life and it is a central factor for pathways involved in cell development and survival, proliferation and renewal. IGF-1 exerts its function by interacting with its receptor IGF receptor 1 (IGF1R; Costales and Kolevzon, 2016). In the CNS, IGF-1 plays a role in growth and development of all major CNS cell types and their synapse maturation. Imbalances in the IGF-1 pathway are associated to neuronal developmental impairment and, in particular, to ASD (Vahdatpour et al., 2016). Indeed, recombinant IGF-1, as well as some related compounds, have emerged as potential therapeutics to treat neurodevelopmental disorders and, indeed, clinical trials for ASD are in progress with these molecules (Wrigley et al., 2017).

In the mouse model of FXS, decreasing the levels of IGF1R corrects a number of phenotypic features (Deacon et al., 2015). Trofinetide is a neurotrophic peptide derived from IGF-1 that shows a long half-life and is well tolerated. A chronic treatment of *Fmr1*-KO mice with trofinetide corrected learning and memory deficits, hyperactivity and social interaction deficits displayed by these animals. At the microscopic level, abnormal dendritic spine density was rescued (Deacon et al., 2015). Considering this promising premise, a phase II clinical trial for trofinetide was performed and after only 28 days of treatment, improvements in higher sensory tolerance, reduced anxiety, better self-regulation and more social engagement were observed. No serious side effects were reported. Interestingly, Neuren Pharmaceuticals reported that trofinetide had significant clinical benefits in a Phase II clinical trial in 5–15 years old girls affected by Rett syndrome, another form of neurodevelopmental disorder characterized by ASD and intellectual disability (Bedogni et al., 2014)^1^.

### Matrix Metalloproteinases Pathway

Matrix metalloproteinases (MMPs) are endopeptidases implicated in both physiological and pathological remodeling of tissues, and their activity is dependent on the zinc ion of their catalytic site. The MMPs family counts 25 members (22 of them found in humans), which can cleave both extracellular matrix (ECM) components and non-ECM elements. MMP-9 is a 92 kD collagenase involved in a broad spectrum of remodeling events of the ECM and plays a major proteolytic role in many cell types. Indeed, it acts during embryo implantation, cardiac tissue development and immune cell functioning (Yabluchanskiy et al., 2013). In the brain, MMP-9 controls synaptic plasticity, and thus learning and memory formation (Ganguly et al., 2013; Knapska et al., 2013).

Until recently, brain disorders associated to defects in MMP-9 has been linked uniquely to its involvement in inflammatory and immune responses. Nevertheless, a hyperactivation of MMP-9 in neurons may cause a massive degradation of the ECM surrounding them that could have severe consequences on function and maturation of synapses. So far, misregulated activation of this enzyme has been implicated in a number of neurodegenerative disorders, including traumatic brain injury, multiple sclerosis and Alzheimer’s disease but also in neurodevelopmental disorders (Reinhard et al., 2015).

The mRNA coding MMP-9 is a target of FMRP, which negatively modulates its expression. Indeed, in the absence of FMRP the expression levels and the activity of MMP-9 are increased. Interestingly this abnormal activity as well as the aberrant dendritic spines could be rescued by treating neuronal cultures with minocycline (Bilousova et al., 2009). This drug is an FDA-approved broad-spectrum antibiotic that shows two major effects: (1) it increases the phosphorylation of GluR1; (2) it promotes the membrane insertion of AMPA receptors (Imbesi et al., 2008). With its action, minocycline lowers the abnormally elevated levels of MMP-9 in FXS, and, when used *in vivo* on *Fmr1*-KO mice, reduces anxiety and reverses the deficit in ultrasonic vocalizations (Rotschafer et al., 2012). Genetic reduction of MMP-9 was obtained by crossing the viable MMP-9 KO mice with *Fmr1*-KO mice. Double KO mice lacked the typical major symptoms of FXS observed in *Fmr1*-KOs (Sidhu et al., 2014).

Until now, minocycline has been shown to be successful in two different clinical trials (Paribello et al., 2010; Leigh et al., 2013) and, importantly, treatment with mynocline resulted in the improvement of some event-related potentials compared with placebo (Schneider et al., 2013). However, the presence of side effects reduces the enthusiasm for the utilization of this molecule in clinic. It is worth to note that the relevance of MMP-9 levels for the physiopathology of FXS has been recently underlined by the rescue of several FXS related behaviors after a chronic treatment of *Fmr1*-KO with metformin that, interestingly, resulted in the reduction of MMP-9 levels in the *Fmr1*-KO mouse brain (Gantois et al., 2017).

### Endocannabinoid Pathway

The endocannabinoid system (eCS) is represented by a group of neuromodulatory lipids and their receptors, notably the cannabinoid receptors 1 (CB1) and 2 (CB2). This system is present in mammalian tissues and, in particular, regulates the cardiovascular, nervous and immune systems. In the brain, the eCS is a key modulator of different neuronal aspects, including synaptic plasticity, cognition, anxiety, nociception and susceptibility to epileptic seizures (Khan et al., 2016). All these features also characterize the FXS patients’ phenotype. Indeed, the lack of FMRP has been associated with impaired functioning of the eCB pathway in glutamatergic synapses, and this identifies the endocannabinoid signaling complex as a possible therapeutic target for FXS (Jung et al., 2012). Rimonabant, a selective antagonist of the CB1 receptor, has been the first drug targeting the eCS used to attenuate the FXS symptoms (Busquets-Garcia et al., 2013). This drug was first developed for the treatment of obesity, but was withdrawn from the market because of its significant psychiatric side effects, such as depression, anxiety and suicidal thoughts. Nevertheless, the adverse effects only appear in patients that were given the highest administered doses. Notably, it was shown that the treatment with very low doses of rimonabant rescues synaptic plasticity in the hippocampus of *Fmr1*-KO mice and also learning and memory (Gomis-González et al., 2016). A new CB1 receptor neutral antagonist (NESS0327) was recently shown to have the same beneficial effects on *Fmr1*-KO mice behavior as rimonabant, supporting the CB1 receptor as a target to treat FXS (Gomis-González et al., 2016).

Deregulation of eCS could be involved in the physiopathology of ASD forms other than FXS. Indeed, 450 children born between 2006 and 2014 from mothers who, during their pregnancy, had taken valproic acid (VPA), an anti-epileptic and mood stabilizing drug showed neurobehavioral dysfunctions. This suggested that VPA plays an important role in developing ASD (Inspection Générale des Affaires Sociales (IGAS), 2016) and subsequently led to the generation of an environmental model of ASD by exposing pregnant rodents to VPA (Williams and Hersch, 1997; Williams et al., 2001). Recently, some social deficits displayed by two VPA-induced ASD rat models have been corrected by treatment with anandamide, an endocannabinoid positively stimulating the eCS pathway (Servadio et al., 2016). These findings further confirm that the eCS may be an interesting and important target for ASD therapy by using both agonist or antagonist approaches.

### Serotonin Pathway

Hyperserotonemia was the first biomarker identified in patients affected by ASD. More recently, it has been shown that the levels of the amino acid tryptophan, the precursor of serotonin, is lower than normal in autistic brains, and that a diet poor in tryptophan worsens autistic symptoms (Boccuto et al., 2013). It was shown that polymorphisms in a gene encoding a 5-HT reuptake transporter protein cause lower synaptic serotonin availability and correlate with increased aggression and destructive behaviors (Hessl et al., 2008). Interestingly, decreased serotonin production was observed especially in young ASD children between 2 and 5 years of age, when the serotonin level should be at its maximal production (Chugani et al., 1999). Animal models in which genes involved in serotonin signaling have been inactivated display altered social interaction (Muller et al., 2016). Conversely, several mouse models of ASD, such as the 15q11-13 duplication and Smith-Laemli-Opitz syndrome models, to which we can also include the Fmr1-KO mouse, display altered 5-HT signaling (Muller et al., 2016). Indeed, it was shown that the stimulation of 5-HT_7_ serotonin receptors in post-synaptic compartments reverses mGluR-LTD in hippocampal slices of FXS mouse brains, suggesting that 5-HT_7_ receptor agonists might be envisaged as novel therapeutic tools for FXS (Costa et al., 2012). These same authors characterized two new molecules with very high binding affinity and selectivity for 5-HT_7_ receptors and ability to rescue exaggerated mGluR-LTD that might be used as novel pharmacological tools for the therapy of FXS (Costa et al., 2015).

The growing body of evidence linking ASD to abnormalities in serotonin function caused the use of the selective serotonin re-uptake inhibitors (SSRIs: citalopram, escitalopram, fluoxetine, fluvoxamine, and sertraline) to target various symptoms of the disorders. Most studies resulted in significant improvements in global functioning and in symptoms associated with anxiety and repetitive behaviors with mild side effects (Kolevzon et al., 2006). Due to the commonalities between ASD and FXS, low-dose sertraline was used to treat young children (12–50 months) affected by FXS. This drug, considered to be one of the most potent inhibitors of serotonin re-uptake, gave significant benefits in behavioral and cognitive features, especially in language skills (Winarni et al., 2012). More recently, a double-blind control trial was performed in 57 FXS patients aged between 2 years and 5 years using another serotonin re-uptake inhibitor (SSRI named Zoloft), an anti-depressant typically used in the treatment of depression, obsessive-compulsive disorder, panic disorder. Despite disappointing primary endpoint results, this treatment demonstrated a positive effect on cognition, visual reception score improving social interaction and early expressive language development (Greiss Hess et al., 2016), strongly suggesting serotonin re-uptake as a promising target to improve the FXS phenotype. Some selective serotonin (5-HT) re-uptake inhibitors seem to act through oxytocin release. Further, the administration of fenfluramine, a serotonergic agonist, to healthy subjects increases plasma oxytocin levels (Marazziti et al., 2012). It is interesting to note that the oxytocin signaling has been proposed as a target to treat FXS (see below). Thus, a better exploration of this cross-talk could result in new therapeutic approaches for FXS and ASD.

### Oxytocin

Oxytocin is a neuropeptide that acts both as a hormone and as a neurotransmitter exerting pleiotropic effects in humans. It is well known to trigger labor, but also induces trust, empathy, and parental-infant relationships. It promotes social behavior and reduces stress and anxiety. In the last decade, it has been shown that intranasal administration of oxytocin is a potential treatment that improves social communication skills in various disorders. Some promising studies in animal models (Meyer-Lindenberg, 2008) paved the way for clinical trials focusing on the treatment of impaired social skills in a variety of conditions, including ASD and schizophrenia. Recent studies suggest that intranasal administration of oxytocin can ameliorate some symptoms of FXS, showing anxiolytic and pro-social qualities (Hall et al., 2012). Ben-Ari et al. (1989) shed light on the molecular mechanisms of the benefits of oxytocin treatment. Oxytocin plays a key role in regulating the effect of GABA on neuron activity. GABA is mostly known as an inhibitory neurotransmitter that acts on a receptor channel complex permeable to chloride anions. These anions flow through the channel according to their electrochemical gradient across the plasma membrane. It is noteworthy that the net value of this gradient changes along the development of the brain, depending on the expression of two major chloride co-transporters (KCC2 and NKCC1) that consequently modify the effects of GABA stimulation (Ben-Ari et al., 1989). A critical period for this shift occurs perinatally and the excitatory-to-inhibitory change of GABA effect is actually mediated by oxytocin receptors (Tyzio et al., 2006).

These authors further reported that the oxytocin-mediated GABA excitatory-inhibitory shift during delivery is abolished in the VPA-treated and *Fmr1*-KO mice, both rodent models of ASD. Consistently, blocking oxytocin signaling in naïve mothers resulted in the production of offspring with electrophysiological and behavioral autistic-like features (Tyzio et al., 2014). These authors also showed that during delivery, both *Fmr1*-KO and VPA-treated mice have elevated intracellular chloride levels in hippocampal neurons. These elevated chloride levels may explain the paradoxical effects of benzodiazapines and phenobarbital that occur through GABA modulation in patients with ASD. Interestingly, the maternal pretreatment with bumetanide, a diuretic belonging to the sulfamyl category that blocks the chloride co-transporter NKCC1, restored electrophysiological and behavioral phenotypes in the offspring (Tyzio et al., 2014). Very recently, the same authors performed a clinical trial and remarkably showed that bumetanide improves the core symptoms of ASD and presents a favorable benefit/risk ratio particularly at 1.0 mg twice a day (Lemonnier et al., 2017). Collectively, these studies strongly indicate that the oxytocin pathway is involved in ASD physiopathology and that bumetanide is a promising treatment for various forms of ASD, including FXS.

## Concluding Remarks

The intense efforts to unravel the physiopathology of FXS appear to have produced some relevant pharmacological targets to treat this disorder. It is interesting to underline that most of the FXS deregulated pathways have been found to be unbalanced also in other forms of ASD or ID-associated diseases. This supports the relevance of these pathways in the physiopathology of ASD and/or intellectual disability. The success of preclinical treatments in mouse, rat or fly FXS models should be considered an exceptionally important result. However, we have learned from past clinical trials that to evaluate the effectiveness of therapies in humans, the design of clinical trials as well as the definition of disease-specific endpoints are of critical importance. Indeed, even on the basis of excellent pre-clinical results, these same preclinical targets have not been translated to therapies to improve behavior and cognition of FXS patients. It is clear that the trials could benefit from the analysis of previous trials results by performing, for instance, longer treatments (Berry-Kravis et al., 2016; Erickson et al., 2017). By further understanding the molecular deregulations in FXS, it should be possible to combine two (or more) drugs targeting different altered pathways, as proposed by the Willemsen laboratory (Zeidler et al., 2015), or treating patients at different ages with different targeted treatments, as we have recently proposed (Bardoni et al., 2017). The positive impact of metformin on FXS behavior troubles underlines the importance to focus on repositioning existing drugs to find new targeted treatments for FXS, as well as for other neurodevelopmental disorders. Lastly, the fact that some clinical trials worked only in small sets of patients (Berry-Kravis et al., 2016), suggests the importance of stratification of FXS patients on the basis of their multiple phenotypes for *“ad hoc”* therapies.

Furthermore, according to Budimirovic et al. (2017), 22 double-blind controlled clinical trials in FXS have been finalized between 2008 and 2015. The accurate analysis of these studies led the authors to the conclusion that the readouts employed to evaluate the outcome of treatments were in general of moderate/poor quality (Budimirovic et al., 2017). In this context, the search for specific and easily measurable biomarkers for FXS should be encouraged. Even if efforts are in progress concerning blood-based and neurophysiological measures (Ethridge et al., 2016; Ray et al., 2016; AlOlaby et al., 2017; Pellerin et al., 2017; Wang et al., 2017), it would be interesting to develop cell-based biomarkers improving analysis of FXS iPS cell lines, while parameters analyzed so far have highlighted the great heterogeneity of these cells (for review see Khalfallah et al., 2017). Also in this case, more detailed studies could result into personalized treatments or treatments concerning subsets of patients. We can underline that only a few examples of FXS cell lines exist that can be used to investigate preclinical treatment *in cellulo* or screening with chemical libraries (Castets et al., 2005; Khalfallah et al., 2017). The availability of such tools could accelerate the definition of new pharmacological approaches identified by the dissection of altered pathways in FMRP-null brains. This dissection can be realized by the analysis of FMRP targets (mRNAs and proteins) and the FXS-translatome in different brain regions and/or neuron subtypes at different ages of neurodevelopment upon different conditions (*e.g*., stress and learning) or upon various stimuli.

All these considerations indicate that research on FXS has still some stimulating areas to investigate in order to define new treatments. In addition, the recent promising studies presented here suggest the conclusion that treatments for all FXS patients will be available in a near future and will not remain only a dream for patients’ families and researchers in the field.

## Author Contributions

SC, BB and TM wrote the manuscript.

## Conflict of Interest Statement

The authors declare that the research was conducted in the absence of any commercial or financial relationships that could be construed as a potential conflict of interest.

## Footnotes

^1^https://www.fraxa.org/tag/trofinetide/
